# Simulations in Cyber-Security: A Review of Cognitive Modeling of Network Attackers, Defenders, and Users

**DOI:** 10.3389/fpsyg.2018.00691

**Published:** 2018-05-15

**Authors:** Vladislav D. Veksler, Norbou Buchler, Blaine E. Hoffman, Daniel N. Cassenti, Char Sample, Shridat Sugrim

**Affiliations:** ^1^DCS Corporation, Human Research and Engineering Directorate, United States Army Research Laboratory, Alexandria, VA, United States; ^2^Human Research and Engineering Directorate, United States Army Research Laboratory, Adelphi, MD, United States; ^3^ICS International, United States Army Research Laboratory, Adelphi, MD, United States; ^4^Vencore Laboratories, Basking Ridge, NJ, United States

**Keywords:** cognitive modeling, behavioral simulations, cyber-security, human factors, model tracing, network simulations, embedded cognition, training effectiveness

## Abstract

Computational models of cognitive processes may be employed in cyber-security tools, experiments, and simulations to address human agency and effective decision-making in keeping computational networks secure. Cognitive modeling can addresses multi-disciplinary cyber-security challenges requiring cross-cutting approaches over the human and computational sciences such as the following: (a) adversarial reasoning and behavioral game theory to predict attacker subjective utilities and decision likelihood distributions, (b) human factors of cyber tools to address human system integration challenges, estimation of defender cognitive states, and opportunities for automation, (c) dynamic simulations involving attacker, defender, and user models to enhance studies of cyber epidemiology and cyber hygiene, and (d) training effectiveness research and training scenarios to address human cyber-security performance, maturation of cyber-security skill sets, and effective decision-making. Models may be initially constructed at the group-level based on mean tendencies of each subject's subgroup, based on known statistics such as specific skill proficiencies, demographic characteristics, and cultural factors. For more precise and accurate predictions, cognitive models may be fine-tuned to each individual attacker, defender, or user profile, and updated over time (based on recorded behavior) via techniques such as model tracing and dynamic parameter fitting.

## 1. Introduction

Computer simulations are of great importance in the field of cyber-security. Simulations are useful as components of network security software and in training exercises for security professionals, as well as software aids designed for network users. Moreover, much of the basic research in cyber-related human factors and cyber epidemiology benefits from simulation software. The dynamics of cyber-security are fundamentally human and adversarial, encompassing a range of attacker, defender, and user interactions. Simulations of human cognition and behavior are of particular importance for addressing these domain characteristics.

Network simulations that include high-fidelity models of users, attackers, and/or defenders may be employed for running wargame training scenarios with realistic traffic and user-generated vulnerabilities. Data collection and analysis from running these simulations provides the means to study how various changes in tools, security restrictions, and training can affect overall network security. Models of users' and defenders' cognition may be employed for real-time estimation of their cognitive states, so as to address human system integration challenges and identify tasks that would benefit from automation. Models of attackers' cognition may be employed in complement with behavioral game theory to predict subjective action utilities and optimal defensive action paths. Just as simulations in healthcare predict how an epidemic can spread and the ways in which it can be contained, such simulations may be used in the field of cyber-security as a means of progress in the study of cyber-epidemiology.

Many of the assumptions made by system administrators and codified as security policies and best practices are based on anecdotal evidence, and are often developed in response to case-studies of prior incidents as part of handling incident response (Boss et al., 2009). Such best-practices are difficult to test empirically and will likely vary depending on the network type and size. Loosening restrictions to see how vulnerable a network becomes in a live setup is irresponsible. Tightening restrictions universally does not always lead to the desired results either, as imposing additional policies and restrictions can prohibit legitimate work and increase the potential for users to stress the network in unintended ways. A simulation of the network and its users, however, provides the ability to test various network policies without real-world consequences. Such simulations may be employed to reveal holes in the procedures and potentially counter-intuitive best-practices.

For example, assumptions for black-listing or white-listing certain websites, port numbers, and software can be examined in the context of realistic models of user behavior and network activity. Even the most conventional of sys-admin wisdom may be based on untested assumptions—such as the idea that certain password complexity requirements increase overall system security. However, several cognitive constraints (e.g., production bias, memory limitation) may force users to cheat by storing passwords in unencrypted text files or employing keyboard visual patterns or other generic patterns (e.g., choosing a password like “asdfASDF1234!@#$”) to more easily recall the passwords. High-fidelity user-models can aid in predicting such behavior, and high-fidelity network simulation can predict how the interaction of restrictions and behavior may affect overall network hygiene. Moreover, testing multiple potential settings can aid in finding a near-optimal configuration for restrictions and other policies.

Current training procedures may have varying effects on different user-types. A high-fidelity cyber simulation should include human users' individual differences. Through such a simulation, we may find that certain training procedures produce healthier overall networks than others. We may produce and begin testing counter-intuitive training regiments, as well (e.g., less training or random schedule training may produce better results for certain user types).

Finally, process models of cognition and behavior can aid in a better understanding of the minds of cyber attackers, defenders, and users, which will further improve network security. Recent research suggests that modeling and predicting attackers' mental state and decisions can lead to better decision aids and a higher rate of thwarted attacks (e.g., Abbasi et al., 2015; Veksler and Buchler, 2016). Defender state of mind is largely ignored in cyber-security tools, although research in cybernetics and automation suggests that cognitive modeling can aid greatly in this domain, as well (e.g., Cassenti and Veksler, 2017). Current best examples of network user behavior predictions are based on statistical analyses to predict common and uncommon timing and locations of access. Computational process models of user cognition can aid in advancing past the common/uncommon classification of user behavior and predict potential errors that lead to security risks. Research suggests that defender/user training effectiveness can benefit from cognitive modeling -based tools, as well.

The rest of this paper discusses the current state of cognitive modeling technology readiness for use in the cyber-security domain. More specifically, the discussion focuses on how specific modeling techniques can be employed in the domain (e.g., model embedding in large-scale network simulations, model tracing, parameter fitting), and outlines prior work that has begun to move the field along these paths.

## 2. The problem of cyber-security

Across organizations and in the literature, “cyber” is a term that reflects a rather large domain. Additionally, the cyber domain overlaps with others, notably the physical (e.g., servers, lines of communication, network topology) and information (e.g., files stored on defended network(s) and servers, control of access to data as per policies) domains. To explore human factors of cyber-security, it is useful to understand the terms and concepts involved and how they impact or are influenced by humans and human behavior.

At the core, security focuses on the CIA (Central Intelligence Agency) triad of confidentiality, integrity, and availability. Information can take on many forms, both digital and physical, with respect to both storage and transmission, and information security must consider the protection of said information and the means with which it is stored and shared. Generally, confidentiality covers the notion of data only being viewed by parties with appropriate permission. It can be considered with respect to the concept of least privilege, wherein any individual only has the privileges absolutely required (permission applied to a user account, for example). The integrity of data means that it is protected from false alteration or corruption when transferred and when stored. Lastly, availability is assurance that the data is accessible by parties with legitimate permission whenever they need it, i.e., without service interruption or unnecessary down time.

However, it is important to recognize that this triad focuses on the security of the data itself, of the IT systems involved. In the networked world, information technology security encompass a wide range of activities and interactions, and human beings are increasingly involved in communication, collaboration, and similar activities that affect and are affected by information security. In reality, security is a process rather than a product (Mitnick and Simon, 2003) that cannot be adequately covered conceptually by the CIA triad. The additional characteristics of authenticity, accuracy, utility, and possession combine with CIA and cover the access paths, ownership, and validity of data across services and organizations to provide a more complete conceptualization of IT security (Whitman and Mattord, 2007). Furthermore, the information security process is not “something that can be bought off the shelf,” requiring an understanding of the people, policies, tools, and techniques involved (Von Solms and Van Niekerk, 2013). Building from the CIA foundation, modern cyber-security is concerned with the tools, policies, concepts, risks management approaches, and best practices that protect information and involved parties from all forms of harm (physical, financial, emotional) that could result from security breaches.

Technical solutions to cyber-security are and should be considered critical to a successful security effort. Employing encryption in communication, access control techniques, and monitoring and auditing tools certainly don't harm security and provide some measure of defense against attacks. However, while tools and policies may provide strong defense for systems and software, human beings can be the greatest vulnerability to security, and human factors must be considered in security perspectives (Jones and Colwill, 2008).

A server may require authentication in order to preserve confidentiality and integrity, but what if a user makes use of a password that is very common, easy to guess, and/or nominally difficult to break? What about a team of employees that have privileged access to machines on the company network that don't require that level of access? In what ways are the employees and members of an organization potential sources of security breaches? Human factors in cyber-security necessitate the need to balance the usability and utility of systems and software with respect to completing work and the policies and limitations placed on them with respect to maintaining security.

Even assuming that perimeter defense tools and techniques, such as firewalls, intrusion detection systems (IDS), filters, and network monitoring methods were completely effective, legitimate uses of a network and its services require communication to be allowed past these walls and boundaries. Human behavior, intentional or otherwise, can form a bridge past these defenses and breach security and are a key area of focus in security policy and practice (Mitnick and Simon, 2003; Jones and Colwill, 2008; Colwill, 2009; Kraemer et al., 2009; Bowen et al., 2011).

Human cognition and behavior is important to understand, model, and predict across many areas of cyber-security. These include attacker-defender game dynamics (e.g., Alpcan and Başar, 2010; Roy et al., 2010), role of deception (e.g., Kelley et al., 2012; Hong et al., 2013; Aggarwal et al., 2016), cyber situational awareness (e.g., Jajodia et al., 2010; Dutt et al., 2013, 2016), and team decision making (e.g., Finomore et al., 2013). Human factors has become a topic of great import to the network security community (e.g., D'Amico et al., 2005; Knott et al., 2013; Mancuso et al., 2014), and one that is specifically focused on predicting and explaining human ability and inability to sift through network traffic data to identify needle-in-a-haystack threats. Importantly, this is an area ripe for contribution via computational cognitive modeling, and, as we will see in the next section, some important headway has already been made in this direction.

Network simulations with high-fidelity behavioral components are of a great interest for professionals seeking to plug these types of breaches. What often arises is a balance between system and network security and the ease with which human actors can complete tasks and achieve their goals on the same systems and networks. The intentions of humans may be at odds with their effects to security. Systems can be configured to impose security on humans, evident in the prevalence of password policies (e.g., character length, character type, and expiration), the management of different levels of account privileges (e.g., not allowing a regular user account to install software, providing a developer with read and write access to a shared repository), and specified configuration of software used in the workplace (e.g., forcing email to display in plaintext and placing restrictions on attachments). Such efforts are means to attempt to provide some foundation of security to build upon across an organization. However, when considering the daily work needs and goals of users, there may be frustration that arises from security impositions. Will humans employ workarounds? Do differing opinions on what is important lead toward management policies that may not support the highest security, such as usability and workflow needs that push against limited access defaults?

In effect, nuances and characteristics of the workplace, motivated by human factors, can chip away at even the most well-intentioned preservation of CIA and cyber-security. Collectively, effects influencing security from internal sources are called insider threats. Insider threat can be both intentional and unintentional. Unintentional insider threat covers instances wherein an administrator perhaps fails to properly configure a server or a well-intentioned user falls victim to a phishing email. Social engineering attacks, while instigated by an external party, can cause employees to inadvertently create insider threat. Intentional insider threat can arise from frustrations or other motivating factors that turn individuals against the organization. External parties may provide the motive through payment, coercion, or political ideology, or an individual may decide to “get revenge” for a perceived wrongdoing or slight. To be best prepared and equipped for cyber-security incidents, an organization must embrace both technical elements of security in design and engineering of systems and networks as well as the cultural and social facets of the humans involved (Colwill, 2009). Ideally, pursuing proper cyber-security practice integrates security knowledge and awareness in the organizational culture as well as system design and implementation. Regardless of the quality of technical cyber-security solutions, analysis of vulnerabilities often covers organizational issues, such as lack of funding, training, or management support, that undermine security efforts and human factors issues, such as poor testing, insufficient communication, and lack of training.

Another area of importance where high-fidelity behavioral simulations are readily employable is training. Training and education are often recommended as an essential method for combating cyber-security vulnerabilities relating to human behaviors and activities within an organization's network (e.g., Verizon, 2017). Beyond human error in configuration or management of a system, awareness of attacker techniques and methods of gaining access, especially those involving social engineering methods and tricking users, can contribute to fewer incidents.

End-user knowledge and education may come in some form of training. For example, purposefully emulating phishing attacks with crafted messages sent to unwitting employees can be used to raise awareness and guide victims to resources and knowledge (Bowen et al., 2011). Virtual environments and games can be used to give participants a consequence-free avenue to explore scenarios and situations related to cyber-security and witness the outcome of their decisions, good or bad (Cone et al., 2007).

## 3. Cognitive modeling in Cyber-Security

There are several ways in which cognitive and behavioral modeling paradigms may be useful in the context of cyber-security. Here we focus on embedded computational process cognitive models and model-tracing techniques. Embedded cognitive models are independent simulations of human cognition and behavior that can interact directly with the task-environment (Salvucci, 2006; Gluck, 2010). In the context of cyber-security, these are cognitive models of network users, defenders, and attackers that can interact with the same software that humans interact with. This may be useful for adding simulated participants in training scenarios, for generating offline predictions in applied tests of network security, or for basic research simulations, especially in the contexts of human-factors and cyber epidemiology.

Cognitive modeling is similar to behavioral modeling, and is often employed for similar purposes. For example, a behavioral model of desktop user behavior may be a Markov state-transition probability matrix, stating that that if the user is in the state where they are typing an email, they may transition to a state where they are looking up something on Google with a probability *x* and a state where they are installing software with a probability *y*. A cognitive model may represent the same state-transitions as state-actions (a.k.a. productions), and assign utilities to each state-action pair. State transitions may be directly calculated based on state-action utilities, with the major difference being that state-action utilities (as well as the states and the actions available in agent memory) will change based on agent experiences.

Simulations of network users, defenders, and attackers require models that include cognitive processes and generic knowledge, as well as domain-specific facts and procedures. There is a variety of cognitive architecture software that attempts to provide modelers with fundamental sets of generic cognitive processes and basic knowledge (e.g., ACT-R, Soar, Sigma, PyIBL, Clarion; Anderson and Lebiere, 1998; Sun, 2006; Anderson, 2007; Laird, 2012; Morrison and Gonzalez, 2016; Rosenbloom et al., 2016). Cognitive architectures often overlap in cognitive theory and capabilities. However, different architectures often have different assumptions and implementations of generic cognitive processes, different modeling languages and requirements, and different level of analysis focus in cognitive time-scale. For this reason, some architectures may be preferable to others depending on the purpose of the modeling effort. For example, Soar and ACT-R architectures both include reward-based learning mechanisms and can update the aforementioned state-action utilities based on agent experiences. However, Soar may be the more appropriate framework for modeling multi-step planning (Laird, 2012), whereas ACT-R may be the better choice when precise fact-retrieval times are of importance (Anderson, 2007).

Regardless of the initial cognitive architecture choice, the modeling system can be tuned based on the specific task and population being modeled. There is no limit to such tuning, enabling modelers to add and remove whole modules in their architecture of choice. However, most of the time such tuning takes the form of parameter value adjustments and model development. Model development is often a form of knowledge engineering—specification of potential goals, inputs, facts, and procedures assumed to be in the mind of the human being modeled.

There are many models simulating parts of network user behavior. For example, in independent efforts Fu and Pirolli (2007) and Peck and John (1992) developed models that make fair predictions as to network user behavior in a web browser based on current goals. There are models simulating how goals are retrieved (e.g., Altmann and Trafton, 2002) and how they are juggled (e.g., Salvucci, 2005). There are user modeling efforts that have focused on social network use (e.g., Hannon et al., 2012), chat behavior (e.g., Ball et al., 2010), team performance (Ball et al., 2010), and email activity (Dredze and Wallach, 2008). Finally, robust models of human cognition, especially in the realm of reward-based motivation (e.g., Nason and Laird, 2005; Fu and Anderson, 2006), can aid in explaining and predicting human behavior in the cyber domain (e.g., Maqbool et al., 2017). There are also many efforts for integrating individual models into a comprehensive model that can encompass multi-agent behavior at network-level dynamics (Romero and Lebiere, 2014). Such models can become an essential component of simulations in cyber, useful for generating realistic traffic and security holes. Model-based agents can act as simulated humans, switching between applications, clicking links, and downloading and installing software.

Attackers and defender models require more domain-specific knowledge. Unfortunately, subject-matter experts in this field are rarely available to the academic groups that do the bulk cognitive model development. Some core components of human-software interaction may be modeled without any deeper understanding of attacker/defender subject-matter expertise. For example, Instance-Based Learning theory (Gonzalez et al., 2003), integrated with memory dynamics of ACT-R (Anderson, 2007), has been employed in efforts to explain situational awareness of cyber analysts (Arora and Dutt, 2013; Dutt et al., 2013; Gonzalez et al., 2014), and to predict the role of intrusion-detection systems on cyber-attack detection (Dutt et al., 2016). These modeling efforts involved abstracted scenarios, but still exemplify useful research for understanding and predicting expert behavior. Moreover, in the case where cognitive models are to be exported as part of decision aid software for real-world cyber-security experts, abstract states and procedures may always be remapped to more specific domain correlates.

Regardless of whether the attempt is to model users, defenders, or attackers, tailoring the model to reflect what may be known about the individuals being modeled may be necessary to achieve better precision and use in the simulation. Model tailoring may be done during and prior to model initialization, as well as live, while the model is running, based on incoming data points. Much of model tailoring takes the form of adjusting model parameters (e.g., learning rate, exploratory tendencies), but some of it takes the form of adjusting model experiences on the fly to match human subject experiences. This latter form of tailoring is known as model-tracing.

The focus of model-tracing is in tuning a cognitive model to real in-task experiences of a specific individual. This technique is employed for maintaining an individual's cognitive state throughout that individual's time within the task-environment. For example, Anderson et al. (1995) employed model-tracing in automated ‘cognitive tutors’ to predict why students made certain errors on algebra problems, so as to better suit instructions to each individual student. In the context of cyber-security, model-tracing of network user and defender cognition can aid in predicting potential biases, errors, and negligence; and model-tracing of attacker cognition can aid in predicting probable attack paths.

The following sections discuss model embedding in network simulations, model initialization and dynamic tailoring, the use of modeling in defender-attacker dynamics, and the use of modeling in automation.

## 4. Standalone model integration in cyber simulations

High fidelity network models are a crucial component to the establishment of effective policy. Since experimentation in production systems is nearly impossible, the need for test environments that can be used to generate reproducible results is apparent. These environments can be used to evaluate policies in existing networks, prototype new networks, and train staff members in a sand-boxed environment where the consequences of mistakes are minimal. Embedding synthetic users, attackers, and defenders in such environments enables evaluations to be of a higher fidelity and, ultimately, accuracy.

### 4.1. Modeling a network

There are three major approaches to consider when modeling a network. The first is simple replication. In this approach we duplicate the existing network (or the relevant parts of it) with another copy of the environment. This approach, under the constraint of a complete replication (all hardware and software is duplicated), yields the best fidelity of modeling, as the test environment is an exact copy of the production environment. This approach has some obvious draw backs. The first of which is cost. A less obvious but more practical concern is the time costs for using the duplicated environment. Because we are working with a physical copy of the production environment, if there are errors in the modifications being tested, re-provisioning the network may be a physical task. This could involve rebooting physical devices and manually reconfiguring hard ware. Depending on the complexity of the environment such a task could take days.

#### 4.1.1. Pure simulation

An alternative method would be to use a purely simulated network. There are many simulators to choose from, including ns-3 (Riley and Henderson, 2010), Opnet (Chang, 1999), and Qualnet (Documentation, 2006), and others (Siraj et al., 2012). While each simulator has its own merit, ns-3 is the most widely used, because it is open source. Since it has such a large community backing the project, the code base is very actively maintained and is well documented. Pure simulation does not suffer from the usage time overhead that a fully replicated network would impose, since it's operation is purely software based. However, it lacks the ability to model real payloads and timings that would be normally present in a regular network. In simulation, all payload data is generated from an assumed distribution. These assumptions may not necessarily reflect the real world traffic distributions. As an example, consider moving a mouse pointer over a modern web browser. As the mouse pointer moves toward a link, the browser may pre-fetch parts of the HTML from the next page to optimize the loading speed of the user's next action. This creates traffic bursts that are erratic and may not necessarily conform to a standard distribution.

Another issue with pure simulation is that it cannot give insight into the behavior of the software that is part of the production environment. In pure simulation, only the network traffic is modeled; each application is represented as a source of network messages (or packets, or frames depending on the layer the simulated network operates at) that require transport. In pure simulation it would not be possible to model the comprise of an operating system by a cyber attack, because there are no operating systems to compromise.

**Figure 1:**
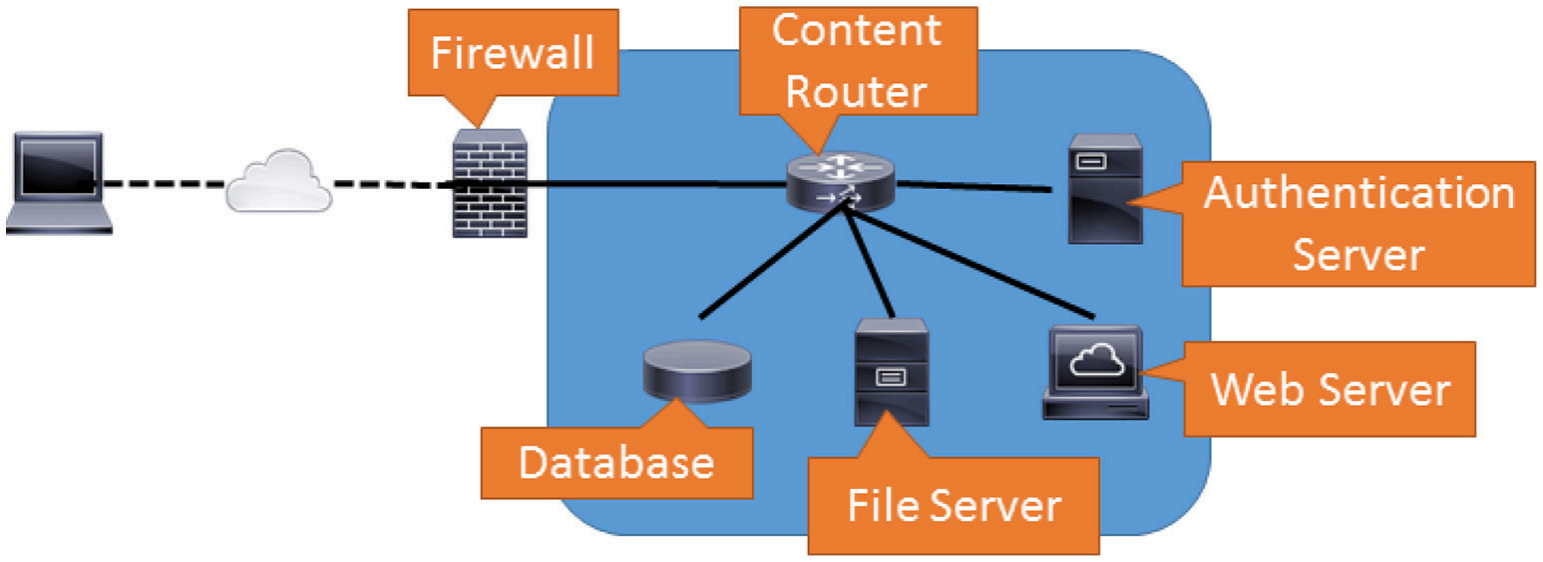
This figure demonstrates how traffic cascades are created in a typical service network. In this network, documents are served to the World Wide Web by coordinating a response amongst several internal services. While the outside user only interacts with the web server, the web server must contact other services on other machines to complete its task. Thus a simple document lookup generates several traffic flows within this network.

Traffic generation in simulation is inadequate, because it cannot model traffic generated by interdependent services. Generators like MGEN (2018)^1^ and IPERF (2018)^2^. simply model traffic flows that originate from a single machine. However, most user-facing network services are the result of several interdependent processes running on networked machines. If we consider the simple example of browsing a web document portal (shown in Figure 1), we can enumerate the cascade of traffic flows that result from a single user's actions.

When the user first browses the web page, the first traffic flow connects the firewall to the web server. This is probably static content and would most likely be stored on the web server. Next the user will need to provide credentials and log in. This procedure will require the web server to contact the authentication server. If the user needs to search for a document, the web server will have to contact the database. Once the document is found, the web server will need to retrieve it from the file server. Even though the user only interacts with the web server, each user action generates multiple flows within the network.

#### 4.1.2. Hybrid network emulation

The final approach, Hybrid Network Emulation (HNE), strikes a balance between the previous two. In a Hybrid network emulator, both the software and network are modeled. The computer hardware is abstracted by using standard machine virtualization techniques such as QEMU-KVM (Habib, 2008) or XEN (Barham et al., 2003). The operating systems (OS) under test are guests in the virtualized environment provided by the hypervisor host (sometimes called the host OS). The network is abstracted with a network simulator such as ns-3.

A key component of this approach is a method for taking the traffic generated by the software running on the virtualized hardware and injecting it into the simulation. The CyberVAN testbed (Chadha et al., 2016), one of the first HNEs, addresses this need by using virtual LAN (VLAN) identifiers as message tags. As a message exits the virtual network interface of a virtual machine (VM) that houses the software under test, it is tagged with a VLAN id that uniquely identifies this traffic to the simulator. The simulator uses this identifier to determine where in the simulated network this traffic should be injected. This tagging operation is critical to the emulation, since a cyber attack on the guest OS means that traffic coming out of the VM might be forged. Thus we can not expect that header information of a message coming out of the virtual interface would reflect the configuration of said interface within the VM host or the guest operating system.

One of the biggest challenges to maintaining the fidelity of the emulated network is timing. The simulated network may require significant computation to determine what to do with a specific message. This is especially true if the simulator is modeling wireless links such as satellite, LTE, or WiFi. Wireless links not only require a delivery decision but also radio channel modeling to determine how long a packet will take to arrive and if any corruption of the packet has occurred. The calculation of the fate of an individual message may take more than one second, and for any given second many messages may be in-flight. It will often be the case that computing what occurs in a simulated second will take significantly longer than one second, thus these simulated networks run slower than real time. Unfortunately, the VMs that are generating this traffic are not inherently aware of this limitation. Because they assume that time is passing according to “wall clock time,” the timers associated with their messages (e.g., TCP session timer) may expire prematurely.

**Figure 2:**
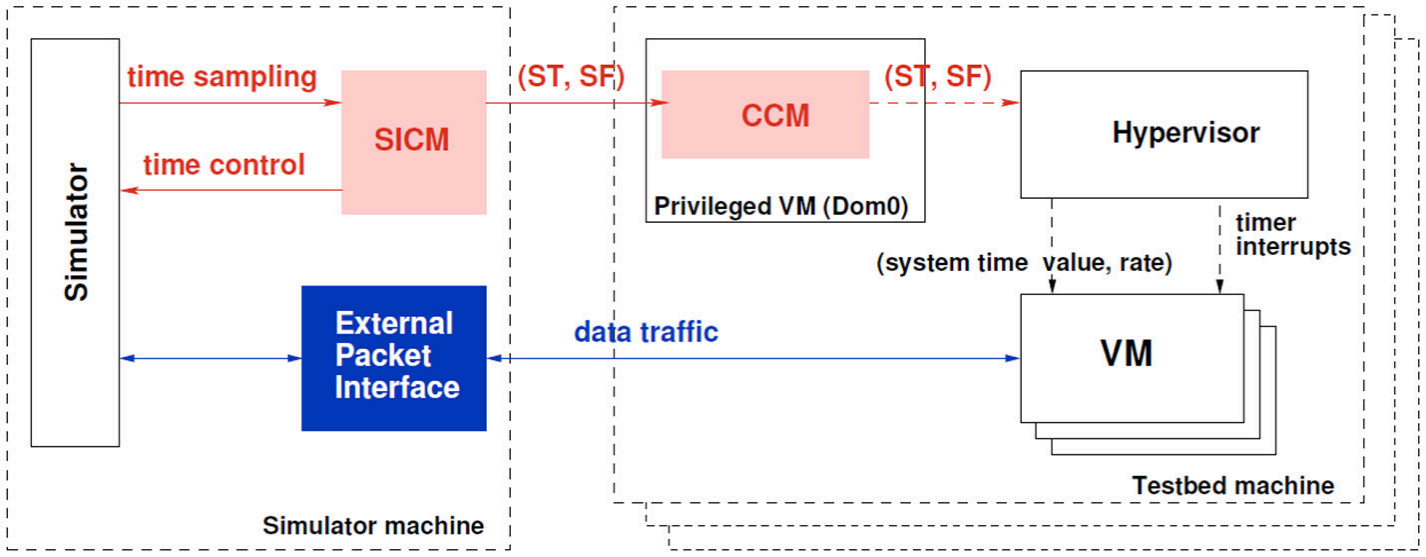
This figure shows architecture of the TimeSync System. While there are many components, the key detail depicted in red is that time information flows from the simulator to the Hypervisor. The Hypervisor presents virtualized hardware to the operating system (e.g., Windows10 or CentOS Linux) which includes system clock information. Thus, the operating system which runs on the hardware is given time information that is adjusted to keep pace with the simulator. If the simulator slows down to handle more complex models, this effectively slows time for the operating system. The internal details of how this is achieved is documented in Sultan et al. (2012).

A solution to this problem is the TimeSync system (Sultan et al., 2012) shown in Figure 2 used in the CyberVAN testbed. Since the VMs are running virtualized hardware and are essentially a software process running on the host OS, the running time of this process can be controlled by the host OS. The TimeSync system adjusts the running time of the VMs to by reacting to timing messages generated by the simulator. The core idea of TimeSync is to create a simulator-driven virtual timeline in the VMs participating in emulation (as opposed to the “real” time line created by the hardware platform that VMs normally follow).

One big advantage of this approach is the ability to instrument both the VMs and the simulated network for measurement. Since real OSes are running on the VMs, standard OS tools like sysdig can be used to collect operating system data. At the same time, the ns-3 simulated network code can be instrumented to enable packet capture on every interface in the entire network. It can also log other information and create digests of flow stastistics. Such a procedure was done to generate the data sets in Bowen et al. (2016).

### 4.2. What can be done with the hybrid network emulator

While hybrid network emulation is not a full replication of a production network, it provides the flexibility of simulation while maintaining the ability to interact with the actual software under test. In the CyberVAN testbed, it is possible to directly interact with the operating systems of the VMs that are part of the emulated network. If some user action or test configuration causes the emulated network to become unusable, the entire emulated network state can be rolled back to a pristine state in a few minutes.

Since all data traffic is going through the simulated network, it is possible to capture traffic at every point in the network. Thus one can trace the propagation of a cyber attack through all the components in the network. While this is possible in a physical replica, it can often be difficult to accomplish in practice due to the limitations of physical hardware (some traffic capture might require the installation of physical network taps).

The CyberVAN testbed currently uses QEMU-KVM as it's hypervisor. This hypervisor can support a large collection of guest OSes and is capable of providing virtual network computing (VNC) access to the virtual console of the VM. The practical application of this capability in the HNE is that it can allow multiple users to interact with the VMs that represent the machines in the emulated network. By leveraging web-based VNC viewers like Guacamole^3^, the testbed can be used to construct a cyber-security test range that allows users to directly interact with the guest OSes on the VMs remotely. Participants in training and competitive exercises can be physically geographically separated. The Guacamole client can be used to record the interactive sessions, thus allowing for collection of user interaction data in addition to network and OS data.

The CyberVAN testbed can also be used in a batch mode where user interaction can be replaced by a scripting infrastructure that kicks off events at specific times (simulated time) during the course of the network simulation. This enables the construction of repeatable experiments which can be run multiple times to validate models and identify anomalous outliers normally only detected by exhaustive searches.

#### 4.2.1. Synthetic users, attackers, and defenders

**Figure 3:**
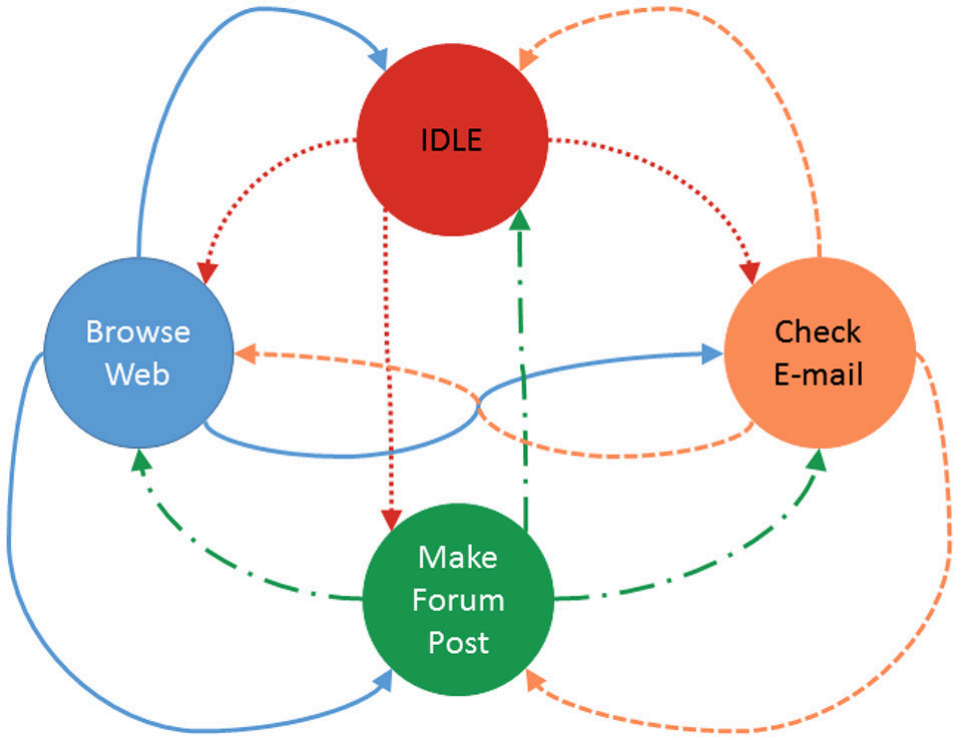
The Markov chain depicted above shows a simplified four state mode of end users behavior. A user starts in the idle state and can then transition to any one of the active states. Each circle is a state that the model will stay in for a time interval (can be varied or fixed). When in an active state, the user will generate some ammount of trafic that is relevant to the state they are in. When the interval ends the next action will be taken based on the probability weights for each transition. More details on the implementation of the Markovbrain that employs this Markov chain can be found in the software's documentation (Renouf, 2017).

The nuances of traffic flows within a network of interdependent services often cannot be modeled by a simple distribution because of the high levels of correlation. Because the VMs in a hybrid network emulator run full operating systems, it is possible to run synthetic users on these virtual machines to generate traffic that is modeled after user behavior. These synthetic users would be most easily implemented as Markov models (see Figure 3) to transition between applications/actions. The operation of the synthetic users proceeds by choosing an interval at random from a distribution that models user activity cycles. When the interval expires, the synthetic user transitions to some other state/activity according to the Markov chain link probabilities.

For example, in the CyberVAN testbed, a synthetic user, named console user (Renouf, 2017), can be added to an experiment to provide realistic background traffic. Instead of simply generating traffic flows in a cycle based on some traffic distribution model, the console user directly interacts with a web browser. The client software initiates queries of a mail server and interacts with a web forum. As noted before, each of these actions can create cascades of traffic flows as the interdependent services communicate to provide the user with the requested service. Thus, for example, traffic flows due to users typing into a submission form are captured.

At best, synthetic user Markov transition probabilities and interval specifications would be based on empirical findings from studies of real network users. However, behavioral transition models are not going to be nearly as informative as embedded stochastic cognitive models that can simulate desktop user behavior, including browsing, downloads, and installs.

Synthetic users present the greatest need for network simulator environments, especially in wargame training scenarios where attackers and defenders are human participants. However, there is also often a need to implement synthetic attackers or defenders of the network. For example, embedded synthetic attackers may be needed for cyber-analyst training scenarios where no attackers are available, or for research studies in cyber-epidemiology.

It is currently the case that on a simulator such as CyberVAN, VMs may be modified to add computational embedded users, or to provide access for human users via VNC. Ultimately, desktop applications could be implemented using some common API (e.g., Veksler et al., 2016) enabling either simulated or real human participants to take control of given VMs and provide symmetric experiences for both real and simulated user-types.

Since a testbed can be instrumented at both the OS level on the virtual machine and in the simulated network, it would be possible to gather data about user, attacker, and defender behavior in the face of specific challenges. Scenarios can be constructed with a specific type of network attack/failure, and user behavior at the VM can be logged as the users attempt to defend the network. This data can be used both to inform best practices about how to handle such attacks and update the models so that they are more effective.

## 5. Model tracing for better predictions of attacker behavior

Game Theory -based decision aids have been used with great success in real-world security contexts (Tambe et al., 2014). However, most approaches to such decision aids have thus far been based on the assumption that human attackers are perfectly rational. Behavioral game theory is a modification of rational game theory informed by, “experimental evidence and psychological intuition” (Camerer, 2003, p. 465). Ultimately, the goal of behavioral game theory is to predict behavior and inform decisions in real-world strategic situations (Gächter, 2008). Whereas the success of normative game theory in security domain comes from providing efficient randomization of security plans and processes, behavioral game theory provides a more realistic view of human strategy selection based on a large body of empirical evidence, and argues for use of behavioral/cognitive models to predict human behavior.

More recent research suggests that modeling and predicting attacker's cognition and action-selection can lead to a higher rate of thwarted attacks. For example, Abbasi et al. (2015) show that employing subjective utilities and prospect theory aids in better predictions of opponent behavior and, thus, better distribution of security resources. Veksler and Buchler (2016) further show that the use of Model Tracing using computational process cognitive models may be employed to dynamically update attacker subjective utilities in repeated game scenarios. When attacker subjective utilities are known, the likelihoods of various attack paths can be directly calculated, and those likelihoods, weighed by the respective risks to the system, can aid in determining the best defensive approach.

Veksler and Buchler (2016) employ model tracing simulations to predict (1) how attacks may be thwarted at a meaningful rate even when much of attacker cognition is unknowable and (2) how slight changes to model parameters based on new behavioral data points can lead to near-optimal prediction of attack paths. In the proposed setup, a simulated attacker bot (sim-attacker) runs on defender-side software, predicting the attacker's most likely actions, and a defender action is suggested based on the attack predicted by this simulation. Once real attacker behavior is observed, the sim-attacker behavior and feedback history are edited to match the observed data, and sim-attacker subjective utilities are updated.

The sim-attacker subjective utilities are updated based on the ACT-R Reinforcement Learning mechanism (Anderson, 2007). The ACT-R model, like any cognitive model, includes parameters that may be adjusted to better reflect the individual or population being modeled. In this particular simulation, these free parameters account for initial biases, learning rates, and exploratory tendencies. Veksler and Buchler (2016) show how parameter values may be adjusted with each new behavioral datum to develop more accurate and precise behavioral predictions. Depending on the network logistics and what is knowable about attacker cognition, these simulations indicate that similar use of cognitive modeling can aid in preventing about 5–30% of attacks over and above what may be accomplished via normative methodology.

## 6. Model tracing for better automation

With the complexity of the tasks they perform, cyber defenders and users are under high mental workload demands (Mancuso et al., 2015). Given both the importance of the tasks, requiring cyber defenders and users to perform well, and that high mental workload demands often lead to decreases in performance, it would be advantageous to provide computerized aids to help with their tasks. The timing of these aids is critical to the benefit they can provide. If computerized aids are constantly active, even when the software user would have been able to perform well without it, the aid can make the user overly reliant on the it (Kaber and Endsley, 2004) and be less discriminating when the aid is inaccurate (Rushby, 2002).

Activating computerized aids at the right time evokes two lines of research: (1) When is the right time to evoke aids? and (2) What aids should be used? Both questions may be addressed by computational modeling (Cassenti and Veksler, 2017). The right time to evoke an aid is based on some analysis of user state. Cassenti et al. (2017) outline three task-concurrent measures that could trigger an adaptive aid, including meta-cognitive (e.g., a button press when the user feels as if help is required), performance-based, and physiological based (e.g., pupil diameter, which had been found to relate to mental workload demand; Naicker et al., 2016). They suggest empirical studies to understand which of these measures are best at determining burgeoning performance declines. No matter which measure is best, determining when to trigger an aid must involve cognitive modeling to understand why performance curves vary over changes in these measures and with different task events. Analyzing cognitive models with model results that have strong statistical relationships to empirical data can provide important insight into the cognitive processing that led to lower performance. Important insights that can be derived may include when there is high cognitive workload, potential for bias-based error, too many competing rules, limited available cognitive resources, or lack of existing relevant knowledge. Cognitive task analyses of new tasks would provide accurate predictions of when similar problems may exist and trigger aids to counteract performance declines.

The other method of investigating adaptive automation with computational modeling is to understand what aids to trigger. Cassenti et al. (2017) analyze cognition into four stages in their descriptive model, including perception, information processing (i.e., sorting and filtering data), decision-making (i.e., selecting a single course of action), and motor control to enact the decision. Cognitive modeling supports the process of analyzing which of these stages is in greatest need of intervention. In a production system type of modeling system, which we recommend (Cassenti and Veksler, 2017), the complexity of stages of a model is determined by such factors as how many mental resources are in operation or the number of mental steps required to complete a cognitive stage. For example, in a model of a cyber defender task with an event feed, a modeler may find that seven mental steps associated with information processing or filtering out unimportant data are required whereas decision making only requires two. In this case, an aid that highlights important elements of the text feed may be more valuable than an aid that attempts to flag single incidents for greater scrutiny.

## 7. Model initialization in the context of cyber

Whether as a part of a full-scale network simulation, or individual-tailored automation, cognitive and behavioral models provide more accurate and precise predictions when tailored to the subpopulation being modeled. Model parameters and knowledge for each model instance should be initiated so as to reflect the mean tendencies of said subpopulations.

Standalone process models may be initiated based on group distributions that may reflect real-world scenarios. In simulations of network users and defenders, we may know a great deal about each person being modeled—e.g., age, gender, education level—and may employ this information in setting up respective models. That is, when a specific user/defender logs on to the network, their information may be used to initiate the simulation. Alternatively, in mass scale simulations, we may be privy to information regarding the distribution of age/gender/skill of our network users.

Attacker models may be more difficult to guess at, though there is a body of research on the correlations between geoip and attacker preferences. For example, cultural values appear to relate to specific attacker behaviors (Sample, 2015; Sample et al., 2016) and preferences (Sample et al., 2017a). Naturally, cyber events do not occur in a vacuum. Cyber is simply the medium used for messaging, and the manner in which cyber is used may be influenced by cultural values.

Sample (2013) first established a link between websites defaced with political messages and citizens of authoritarian, collectivist countries. This led to a study (Sample, 2015) that examined these defacements in the context of kinetic events (e.g., geopolitical disputes). The findings of the 2015 study revealed the strongest association between website defacements and kinetic events occurred with attacker societies that had authoritative, confrontational, competitive, and restrained values (Hofstede et al., 2010) in common (Sample, 2015). In fact, restrained cultural values appeared as the greatest predictor of cyber responses to kinetic events.

Furthermore, the view of a single cyber attack culture, aka “hacker culture,” that follows an international playbook such as top attacks as listed by widely known Internet security organizations was called into question in 2016 (Sample et al., 2016). In the 2016 exploratory study Sample et al. observed certain cultural values associating with specific attack method (a.k.a. attack vector) preferences. For example, short-term oriented cultural values appeared to associate with the use of 0 day attacks as a method to deface a website. In that same study, URL poisoning appeared to be the attack vector preferred by attackers from culturally restrained societies.

The aforementioned studies led to a follow-on study comparing attack vectors of self-identitied attackers (Sample et al., 2017a). This most recent study examined 7 different attack vectors over a 10-year period to determine if cultural values associate with attack vector preferences. This study relied on an examination of seven different attack vectors that were consistent with the groupings enumerated and defined in MITRE's CAPEC groupings for attacks patterns. The results showed different attack vector preferences that coincide with cultural values. Attacks that are bold in nature and leave a significant amount of evidence (e.g., Brute force attacks) were favored by those cultures with masculine values. While attacks that require precision and are more difficult to detect, they appear to be favored by attackers who are uncomfortable with the new or unknown, implying that this group of attackers may engage in more thorough planning with less risk taking when compared to other hackers.

Collectively analyzed, these studies reveal that the way that cyber actors view and interact with the virtual environment is as varied as their interactions and vision of the physical environment. Thus, when examining cyber attackers distinct differences exist, and cultural values appear to be one way to organize this phenomenon. Based on the previously mentioned studies that showed differences in attacker preferences that associated with common cultural values, a logical question would be as follows: do cyber defenders and cyber victims also share cultural value commonalities?

Cyber Victims and defenders appear to be less studied than their attacker counterparts. A study by Sample and Karamanian (2015) suggested that cyber defense strategies differ based on cultural values. In this study a higher adoption rate of the Domain Name System security extensions appeared to be favored by countries with long-term oriented, egalitarian cultural values. Thus, much like in the physical world, defensive behaviors in the virtual world appear to be shaped in part by cultural values.

Most recently Sample et al. (2017b) examined over 17,000 records of social engineering attack victims found in the Zone-H archives spanning 4 years. The victims of social engineering attacks tended to be from individualistic, egalitarian, long-term oriented societies. While the findings from this particular study may lead some to draw conclusions about victims and victim blaming, the real value lies in learning what types of attacks may be most effective against different groups of people due to that group's cultural values and how those values serve to empower users in the cyber domain. These conclusions are rather relevant now that the targets are both more numerous and more diverse.

Naturally, cyber events do not occur in a vacuum. Cyber is simply the medium used for messaging, and the manner in which cyber is used may be influenced by cultural values. In this way finding out geo-ip information can lead to establishing many initial model parameters, procedures, and biases for attacker models. Given the specific target network, we may have additional knowledge about likely attack origins, age, education-level, intent, and gender of hackers, which may all aid in further fine-tuning cognitive models and produce better predictions. Finally, to re-iterate, model tuning may be done at any point in the lifetime of the model, such that as known factors regarding attackers change, the model may be changed immediately.

## 8. Conclusions

Cyber-security is ultimately the interaction of human cognition and adversarial behavior in the context of computer networks. Simulations of human cognitive processes can be of great use for simulating and predicting user error and negligence, defender best-practices, most likely attack behavior, and ultimately, network vulnerabilities. Such simulations/predictions may be useful for training, decision-aid software, and basic research in cyber-security.

Specifically, we describe the use of cognitive models as embedded computational agents for simulating human interactions with software and networks, and the use of cognitive models in the context of model-tracing for keeping track of human cognitive states to make better predictions of potential decisions and biases. The former use-case employs high-fidelity cognitive process models as agents that have access to desktop software via keyboard/mouse control or standard API. In this way we may simulate human use and abuse of the network and predict effects of software use, firewall setup, training, and potential policy changes. Simulating users (and potentially attackers) on the network additionally provides realistic network traffic and vulnerabilities for cyber training/wargame scenarios.

The latter use-case focuses on matching the experience of a specific individual in-task to model experience. In this way the model can trace the cognitive state of that specific user, defender, or attacker at every step. In the case of tracing attackers this becomes useful for predicting and counteracting likely and especially harmful attack paths. In the case of tracing users and defenders this is especially useful for sensing potential overload and error and triggering automation.

Regardless of the use-case, cognitive models provide more accurate and precise predictions of behavior when tailored to the subpopulations being modeled. Model parameters, biases, and known facts and procedures will all differ depending on factors such as age, education level, and, of course, network intent. An internal survey could reveal relevant details regarding users and defenders of a specific network, and geo-ip information in combination with information as to the likely attack-origins may be employed to tailor model software for better attacker predictions.

The use of high fidelity tailored computational process cognitive models of network users, defenders, and attackers can provide accurate simulations that may be useful in cyber-security research and applied contexts. Predictions of behavior may be used in decision-aid software for defenders that will directly impact network security, for dynamic estimates of individual-tailored training requirements, and for predicting likely attack paths. Process models enable development of realistic synthetic users for full-scale training/wargame scenarios. Finally, such models enable much-needed research in cyber-security and cyber-epidemiology.

## Author contributions

All authors listed have made a substantial, direct and intellectual contribution to the work, and approved it for publication.

### Conflict of interest statement

Author SS was employed by company Vencore Labs. All other authors declare no competing interests.

## Footnotes

^1^Available online at: http://mgen.pf.itd.nrl.navy.mil

^2^Available online at: http://software.es.net/iperf/

^3^Apache Guacamole. Available online at: https://guacamole.incubator.apache.org/
